# Delineation of renal protein profiles in aristolochic acid I-induced nephrotoxicity in mice by label-free quantitative proteomics

**DOI:** 10.3389/fphar.2024.1341854

**Published:** 2024-05-09

**Authors:** Xinhui Liu, Shanshan Wu, Yu Peng, Liwen Gao, Xi Huang, Ruyu Deng, Jiandong Lu

**Affiliations:** ^1^ Department of Nephrology, Shenzhen Traditional Chinese Medicine Hospital, Guangzhou University of Chinese Medicine, Shenzhen, Guangdong, China; ^2^ The Fourth Clinical Medical College, Guangzhou University of Chinese Medicine, Shenzhen, Guangdong, China; ^3^ Shenzhen Traditional Chinese Medicine Hospital Affiliated to Nanjing University of Chinese Medicine, Shenzhen, Guangdong, China

**Keywords:** aristolochic acid I, nephrotoxicity, aristolochic acid nephropathy, proteomics, label-free

## Abstract

**Introduction:** Aristolochic acid nephropathy (AAN) is a kidney injury syndrome caused by aristolochic acids exposure. Our study used label-free quantitative proteomics to delineate renal protein profiles and identify key proteins after exposure to different doses of aristolochic acid I (AAI).

**Methods:** Male C57BL/6 mice received AAI (1.25 mg/kg/d, 2.5 mg/kg/d, or 5 mg/kg/d) or vehicle for 5 days.

**Results and discussion:** The results showed that AAI induced dose-dependent nephrotoxicity. Differences in renal protein profiles between the control and AAI groups increased with AAI dose. Comparing the control with the low-, medium-, and high-dose AAI groups, we found 58, 210, and 271 differentially expressed proteins, respectively. Furthermore, protein-protein interaction network analysis identified acyl-CoA synthetase medium-chain family member 3 (Acsm3), cytochrome P450 family 2 subfamily E member 1 (Cyp2e1), microsomal glutathione S-transferase 1 (Mgst1), and fetuin B (Fetub) as the key proteins. Proteomics revealed that AAI decreased Acsm3 and Cyp2e1 while increasing Mgst1 and Fetub expression in mice kidneys, which was further confirmed by Western blotting. Collectively, in AAI-induced nephrotoxicity, renal protein profiles were dysregulated and exacerbated with increasing AAI dose. Acsm3, Cyp2e1, Mgst1, and Fetub may be the potential therapeutic targets for AAN.

## 1 Introduction

Aristolochic acids (AAs), a family of phytochemicals with carcinogenic, mutagenic, and nephrotoxic properties, are common contained in Aristolochia and Bauhinia plants and have been found all over the world (Yang et al., 2018). AAs will induce aristolochic acid nephropathy (AAN), a rapidly progressive interstitial nephritis that often results in acute kidney injury (AKI) and ultimately leads to end-stage kidney disease or urothelial malignancies (Shibutani et al., 2007). Previous studies demonstrated that experimental AAN is characterized by transient acute proximal tubule necrosis, inflammatory cell infiltrates, interstitial fibrosis, and tubular atrophy (Chen et al., 2022). Drugs derived from Aristolochia species have been widely used in many countries for a long time (Heinrich et al., 2009), and there are also areas where crops are contaminated with aristolochic acid (Jelaković et al., 2019). People may be consciously or unintentionally exposed to AA through diet or therapy, which puts plenty of people at risk of the disease. The progressive lesions and mutation events caused by AA are irreversible, and there is no effective treatment for AAN. Therefore, it is urgent to further clarify the molecular and cellular mechanisms of AA-induced nephrotoxicity (Luciano and Perazella, 2015).

Mass spectrometry (MS)-based proteomics has become a powerful and systematic method for large-scale protein analysis (Giansanti et al., 2022). It is a powerful tool for detecting targets in response to drug therapy/toxicity by monitoring changes in protein expression, and the strategy for discovering targets is to compare differentially expressed proteins (DEPs) in biological samples, which reflect the progress of *in vivo* toxicity or are used as prognostic markers (Titz et al., 2014). Recently the method is more and more widely used to explore the pharmacological and toxicological mechanisms of herbs (Bennett and Devarajan, 2018). Label-free quantitative method is one of the important applications in the field of proteomics research. Its basic principle is based on the peak area of peptide parent ion extraction of extracted ion current to identify the peptide and protein in the sample, and then quantify the identified peptide (protein) (Anand et al., 2017). However, there are few proteomics reports on the toxicity of AAI, and previous studies have generally used one dose. While it is rare to intake a high dose one-time, so that the effects of different doses on the kidney remain to be explored.

In this study, we used label-free proteomics technology combined with bioinformatics analysis to delineate renal protein profiles in AAI-induced nephrotoxicity in mice and identify the key proteins affected by AAI in the kidney of mice. It is expected to identify nephrotoxic targets for AAI and deepen our understanding of the mechanism of AAN to reduce the global burden of it by developing novel treatments.

## 2 Material and methods

### 2.1 Animals and experimental protocol

Male 6-week-old C57BL/6 mice (weighing 20 ± 2 g) were provided by the Guangdong Medical Laboratory Animal Center (SCXK (YUE) 2018-0002, Foshan, China). The mice were housed under controlled conditions of light (12 h light/dark cycle), temperature (24°C ± 2°C) and humidity (50%–60%) and had adequate food and tap water to *ad libitum*. The mice were divided into four groups randomly after 1 week of acclimatization: C, control group (n = 6); L, low-dose AAI group (n = 6); M, medium-dose AAI group (n = 6); H, high-dose AAI group (n = 6). Mice in the AAI groups were administered by intraperitoneal injection of AAI (A5512, Sigma-Aldrich, St Louis, MO, United States) at the dose of 1.25 (L), 2.5 (M), and 5 mg/kg/d (H), respectively, for 5 days. Control mice were injected intraperitoneally with PBS solution containing 5% DMSO as vehicle for 5 days. Twenty-4 hours after the last dose of intraperitoneal injection of AAI or vehicle, all mice were euthanized. Blood and kidney samples were collected immediately for further experiments. All animal experiments were carried out in accordance with the National Research Council’s Guide for the Care and Use of Laboratory Animals and approved by the Ethics Committee of Shenzhen Top Biotech Co., Ltd (approved ID: TOP-IACUC-2021-0137).

### 2.2 Serum biochemical analysis

The mice serum was prepared by centrifuging blood for 10 min at 2,000 rpm. Then, the supernatant was transferred to a new clean EP tube. The levels of serum creatinine (SCR) and blood urea nitrogen (BUN) were measured using commercially available kits (SKT-217 and SKT-213, StressMarq Biosciences, Victoria, British Columbia, Canada), according to the manufacturer’s protocols.

### 2.3 Histopathological examination

The upper poles of mice kidneys were fixed in 4% paraformaldehyde overnight, dehydrated and embedded in paraffin. For periodic acid-Schiff (PAS) staining, kidney wax blocks were cut into 4 μm sections, dewaxed, and rehydrated. Then the sections were oxidized in 0.5% periodic acid solution for 5 min. After rinsing in distill water, the sections were placed in Schiff reagent for 15 min and washed in lukewarm tap water for 5 min. The nuclei were counterstained with hematoxylin. Kidney lesions were scored by estimating the percentage of tubules in the cortex exhibiting tubular atrophy, tubular dilatation, vacuolar degeneration, loss of brush border, and cell necrosis and shedding with the following manner (0-5 points): 0 = none; 1 = ≤ 10%; 2 = 11–25%; 3 = 26–45%; 4 = 46–75%; 5 = ≥ 76% (Cortes et al., 2018). Three mice from each group were blindly assessed for tubular injury score using three microscopic fields (×200) per mouse.

### 2.4 Label-free quantitative proteomics and bioinformatics analysis

#### 2.4.1 Protein digestion

The kidney cortical samples were homogenized in lysis buffer consisted of 2.5% SDS/100 mM Tris-HCl (pH 8.0). Then the samples were subjected to treatment with ultra sonication (30%–35%, 10 min). After centrifugation (12,000 g, 5 min), 4 times the volume of precooled acetone was added to precipitate the protein in the supernatant. The protein pellet was dissolved in 8M Urea/100 mM Tris-Cl. After centrifugation (12,000 g, 5 min), the supernatant was used for reduction reaction (10 mM dithiothreitol, 37 °C for 1 h), and followed by alkylation reaction (40 mM iodoacetamide, room temperature/dark place for 30 min). Protein concentration was measured by Bradford protein assay kit (C503031, Sangon Biotech, Shanghai, China). Using 100 mM Tris-HCl (pH 8.0) to dilute urea until it below 2 M. Trypsin was added at a ratio of 1:50 (enzyme: protein, w/w) for overnight digestion at 37°C. The next day, trifluoroacetic acid was used to bring the pH down to 6.0 to end the digestion. After centrifugation (12,000 g, 5 min), the supernatant was purified by Sep-Pak C18 desalting column (Labpart, Beijing, China). Desalination steps were as follows: (1) Activation: add 30 µL ACN to the desalination column, centrifuge at 3,000 g for 1 min; (2) Equilibrium: add 30 µL 0.1% TFA to the desalination column, centrifuge at 3,000 g for 1 min; (3) Sample loading: load the sample into the column after equilibrium, centrifuge at 3,000 g for 2 min at a time of 50 µL until the sample was completely loaded; (4) Wash: add 30 µL 0.1%TFA into the desalination column, centrifuge at 3,000 g for 1 min; (5) Repeat step (4) twice; (6) Elution: change tubes, add 10 µL 80% ACN/0.1% TFA to desalination column, centrifuge at 3,000 g for 1 min. The peptide eluate was vacuum dried and stored at −20 °C for later use.

#### 2.4.2 Liquid chromatography-tandem mass spectrometry (LC-MS/MS) analysis

LC-MS/MS data were collected using a Q Exactive Plus mass spectrometer in series with an EASY-nLC 1200 system. The peptide samples were dissolved in the loading buffer, then inhaled by the autosampler and combined to the analytical column (50 μm * 15 cm, C18, 2 μm, 100 Å) for separation. An analytical gradient was established using two mobile phases (mobile phase A: 0.1% formic acid and mobile phase B: 0.1% formic acid, 80% acetonitrile). The flow rate of the liquid phase was set at 300 nL/min. Mass spectrometry data were collected in data dependent acquisition mode, and each scan cycle contained one MS full scan (R = 70 K, AGC = 3e6, max IT = 20 ms, scan range = 350–1800 m/z) and 15 subsequent MS/MS scans (R = 17.5 K, AGC = 2e5, max IT = 50 ms). High energy collision dissociation was set to 28. The screening window of the quadrupole was set to 1.6 Da. Former target ion exclusion was set for 35 s.

#### 2.4.3 Protein identification and data analysis

Mass spectrum data were retrieved by MaxQuant (V1.6.6) software using the database retrieval algorithm Andromeda. The database used for the search was Swissprot.Mouse.20200826.fasta Proteome Reference Database. The main search parameters were as follows: LFQ project type; Oxidation (M), Acetyl (Protein N-term), Deamination (NQ) were selected for variable modification; Carbamidomethyl (C) was selected for fixed modification; Trypsin/P was selected for enzyme digestion; the matching tolerance of primary mass spectrometry was set to 20 ppm in the initial search and 4.5 ppm in the main search; the matching tolerance of secondary mass spectrometry was set to 20 ppm; check “match between runs.” The search results were screened based on 1% FDR at protein and peptide levels, and anti-library proteins, contaminating proteins, and protein entries with only one modified peptide were deleted. The remaining identification information was used for subsequent analysis. DEPs were defined as fold change greater than 2 or less than 0.5, *p*-value less than 0.05. DAVID database (v2023q2, https://david.ncifcrf.gov) was used for bioinformatics analysis, including Gene Ontology (GO), Kyoto Encyclopedia of Genes and Genomes (KEGG) and Clusters of orthologous groups for eukaryotic complete genomes (KOG). The protein-protein interaction (PPI) network of DEPs was established using the search tool for interacting genes (STRING) database (V12.0, http://www.string-db.org/). Then the PPI network data visualization was realized by Cytoscape (V3.9.1). Principal component analysis (PCA) analysis was performed using the built-in statistical prcomp function of R software (base package, V3.5.1). Set prcomp function parameter scale = True, which means UV (unit variance scaling) processing is performed on the data, and the calculation is based on the Euclidean distance between samples. Heat maps were drawn with R (pheatmap, V1.0.12).

### 2.5 Western blotting

The frozen kidney cortex (approximately 20 mg) was pulverized in liquid nitrogen and resuspended and homogenized in 400 μL RIPA lysis buffer (#9806, Cell Signaling Technology, Beverly, MA, United States) containing a protease-phosphatase inhibitor cocktail (A32961, Pierce, Rockford, IL, United States). The homogenate was centrifuged at 12,000 rpm for 10 min at 4°C, and the supernatant was collected for quantification by Bradford protein assay kit (C503031, Sangon Biotech, Shanghai, China). Equal amounts of protein were separated on 10% SDS-PAGE and subsequently transferred onto polyvinylidene difluoride membranes by electroblotting. Electrophoresis conditions: Tris-Glycine SDS Running Buffer, 90–120 V, 2 h. Electrotransfer conditions: Tris-Glycine Transfer Buffer, 90 V, 1.5 h. After being blocked with non-fat milk for 1 h at room temperature, these membranes were incubated with primary antibodies overnight at 4°C. The primary antibodies against fetuin B (Fetub, 67002-1-ig), cytochrome P450 family 2 subfamily E member 1 (Cyp2e1, 19937-1-AP), acyl-CoA synthetase medium-chain family member 3 (Acsm3, 10168-2-AP) and glyceraldehyde-3-phosphate dehydrogenase (GAPDH, 60004-1-IG) were purchased from Proteintech (Wuhan, China). Anti-microsomal glutathione S-transferase 1(Mgst1, ab131059) was purchased from Abcam (Cambridge, MA, United States) and anti-β-actin (A5441) was from Sigma-Aldrich (St Louis, MO, United States). Horseradish peroxidase (HRP)-conjugated anti-mouse IgG (SA00001-1) obtained from Proteintech (Wuhan, China) and HRP-conjugated anti-rabbit IgG (#7074) obtained from Cell Signaling Technology (Beverly, MA, United States) were used for secondary antibody incubation at room temperature for 1 h. Immobilon^®^ ECL Ultra Western HRP Substrate (WBULS0500, Merck, Billerica, MA, United States) working solution was prepare by gently mixing solutions A and B in a 1:1 ratio and was used to incubate the blots for 2 min at room temperature. Finally, immunoblotting was observed, and image densitometry analysis was performed by Image Lab™ software (Bio-Rad Laboratories, Hercules, CA, United States).

### 2.6 Statistical analysis

Statistical analysis and graphing were performed using GraphPad Prism 9 software (GraphPad Software, Inc., San Diego, CA, United States). The significance of the differences among groups was examined by one-way analysis of variance (ANOVA) followed by *post hoc* analysis with Dunnett’s test. Data are presented as mean ± standard error of the mean (SEM). *p*-value less than 0.05 was considered statistically significant.

## 3 Results

### 3.1 AAI caused nephrotoxicity in mice

**FIGURE 1:**
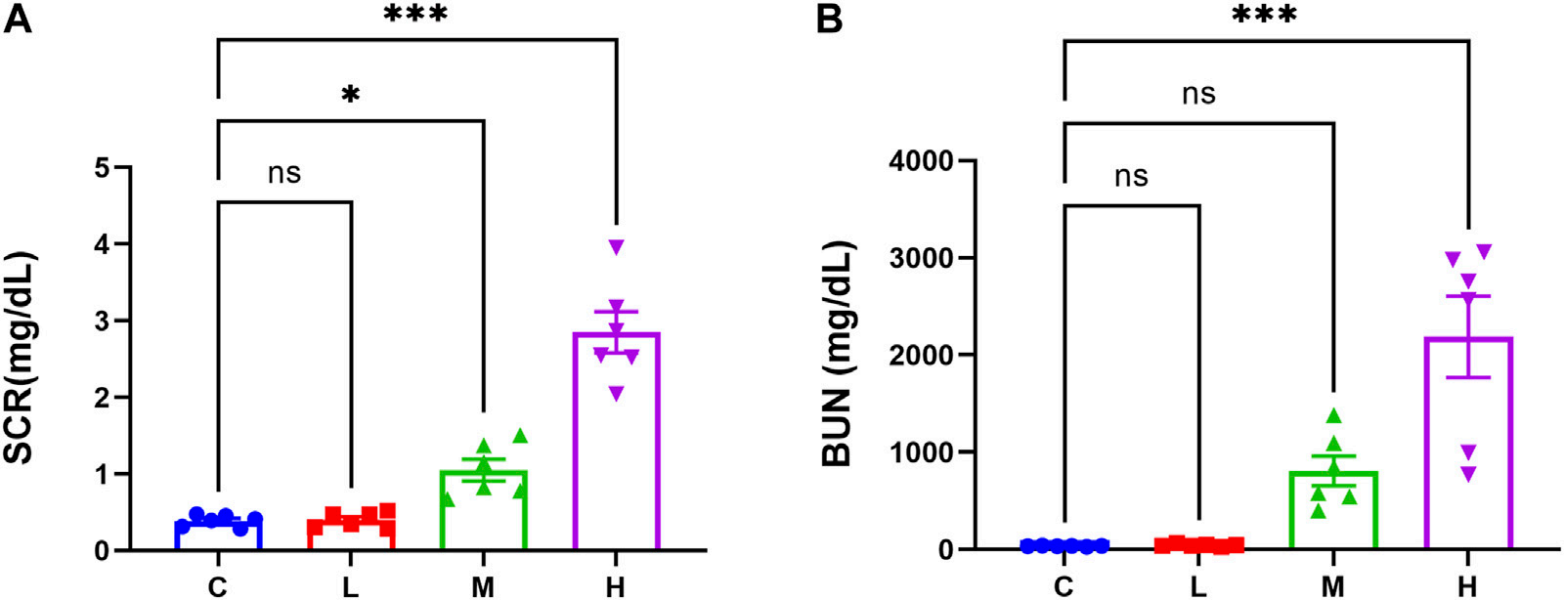
Effects of AAI on renal function in mice. **(A)** Serum creatinine levels (n = 6). **(B)** Blood urea nitrogen levels (n = 6). Data are expressed as mean ± SEM (*p < 0.05 and ***p < 0.001 as compared to the control group).

**FIGURE 2:**
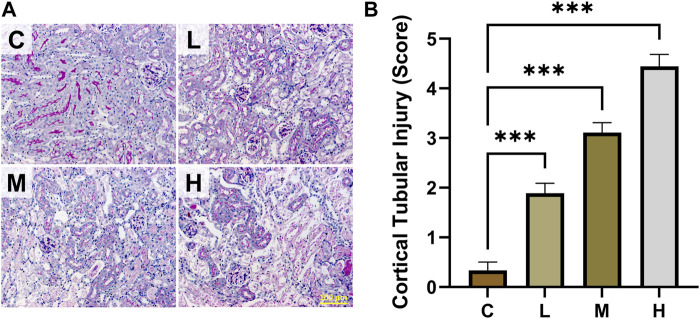
Effects of AAI on renal pathological injury in mice. **(A)** Representative PAS staining images. **(B)** Cortical tubular injury scores (n = 3). All images are shown at identical magnification, ×200, scale bar = 100 μm. Data are expressed as mean ± SEM (***p < 0.001 as compared to the control group).

The results of kidney function-related indexes in mice are shown in Figures 1A, B. Both SCR and BUN levels were considerably higher in the high-dose AAI group than in the control group (*p* < 0.001). The medium-dose AAI group showed a significant increase in SCR (*p* < 0.05) compared with control, while no significant difference was observed in the low-dose AAI group. Furthermore, no significant difference was found in BUN levels in low- and medium-dose AAI groups in contrast to control. In PAS staining, the control group showed clear structure of renal cortex and tight arrangement of renal tubules (Figure 2A). On the contrary, in the AAI groups, necrosis and exfoliation of proximal convoluted tubular epithelial cells were observed along with dilation of tubular lumen, vacuolar degeneration, and infiltration of inflammatory cells in certain areas (Figure 2A). Pathological injuries were scored according to the scoring criteria mentioned above (Figure 2B). These data indicated that the degree of kidney injury in mice was positively correlated with the intake of AAI.

### 3.2 Quality control for proteomics analysis

To improve the quality of proteomics analysis results and reduce the false positive rate, the quality control of the screening results was carried out. Peptides with too short sequences (less than 7 amino acids) are mostly filtered out due to their simple composition, while peptides with too long sequences (generally higher than 40 amino acids) are difficult to be identified by mass spectrometry due to their high molecular weight. Most peptides in this study were in the range of 7–20 amino acids in length (Supplementary Figure S1A). The distribution of the number of missed cut sites of peptides reflects the completeness of enzymatic cleavage. The results showed that the peptide with 0 missed cleavage number was the most abundant, which indicated that the enzymatic cleavage was complete and favorable for identification (Supplementary Figure S1B). The mass deviation of the peptide m/z was normally distributed in the range of −10 to 10 ppm, indicating the ideal mass accuracy of the mass spectra (Supplementary Figure S1C). Moreover, no contamination (marked red) was found in the analysis (Supplementary Figure S1D). Collectively, these quality control data ensured the accuracy of the results of this study.

### 3.3 Protein annotations

After the screening of the above conditions, we obtained a total of 4,141 proteins. To investigate the function of proteins, functional database annotation was made on the identified proteins. The proteins mainly localized in the cytoplasm (30.99%), nucleus (20.56%), and mitochondria (15.53%) (Supplementary Figure S2A). Domains are the basic units of protein structure, function and evolution. The most annotated domain was P-loop_NTPase (Supplementary Figure S2B). In GO annotation, integral component of membrane, protein transport, and adenosine triphosphate (ATP) binding was the most annotated entry in cellular component (CC), biological process (BP), and molecular function (MF), respectively (Supplementary Figure S2C). In KEGG annotation, the metabolic pathways were the most enriched pathway (Supplementary Figure S2D). In the KOG database, the top 3 protein functions were general function prediction only, signal transduction mechanisms, and posttranslational modification, protein turnover, chaperones (Supplementary Figure S2E).

### 3.4 Protein expression

**FIGURE 3:**
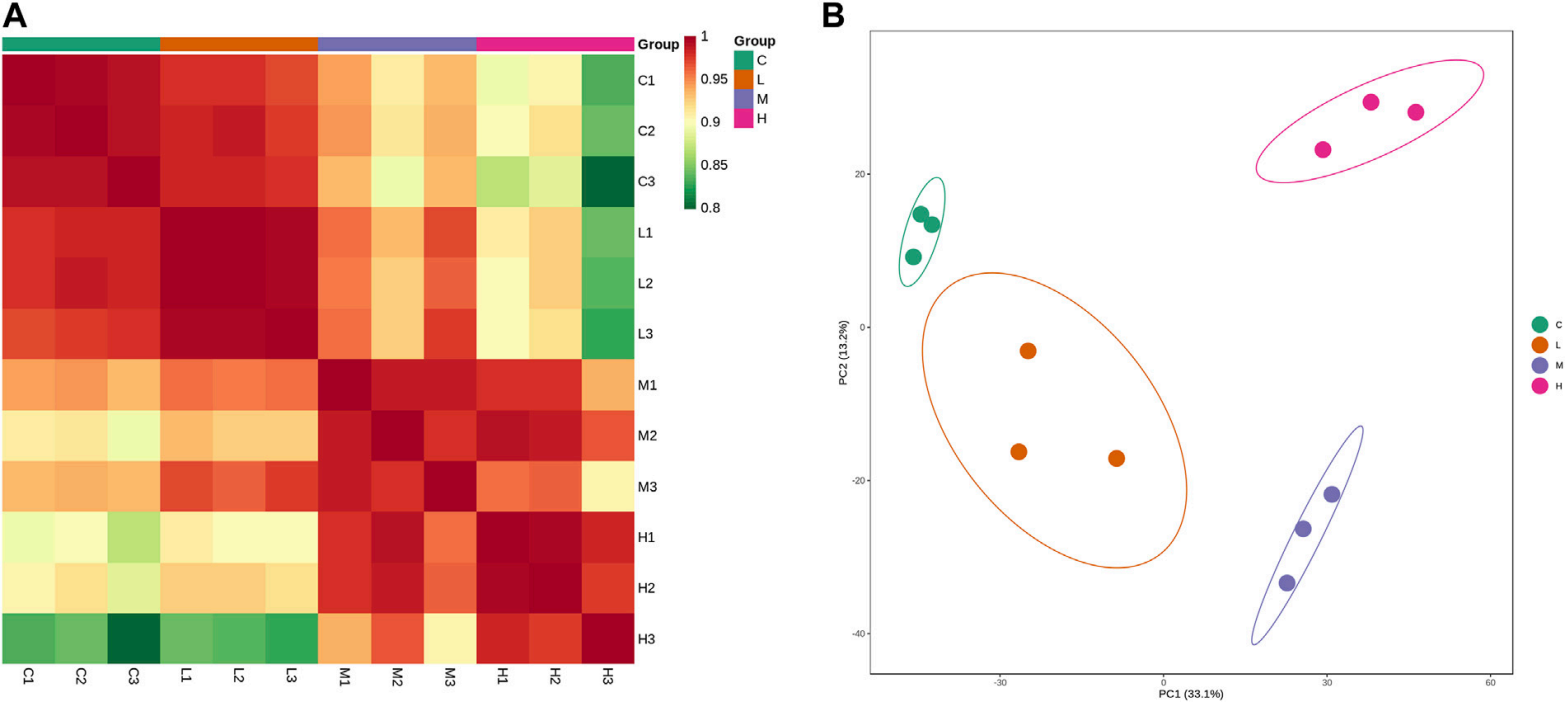
Protein expression analysis. **(A)** Correlation analysis of protein expression of each sample. **(B)** Principal component analysis. Each dot in the graph represents a sample, green represents the control group, orange represents the low-dose AAI group (1.25 mg/kg/d), blue represents the medium-dose AAI group (2.5 mg/kg/d), and pink represents the high-dose AAI group (5 mg/kg/d).

A total of 3,802 proteins with relative quantitative values were further subjected to expression analysis. In Figure 3A, correlation analysis revealed a high correlation of protein expression between the three samples within each group, which indicated good biological replication between the samples within the group. Compared with the control group, the correlation of protein expression between groups became smaller as the AAI dose increased. This suggested that the more severe the AAN model the greater the difference in renal protein expression, with the most pronounced difference between the control and high-dose AAI (5 mg/kg/d) group (Figure 3A). Principal component analysis confirmed that protein expression differences between groups were amplified with increasing AAI dose (Figure 3B).

### 3.5 Identification of DEPs

**FIGURE 4:**
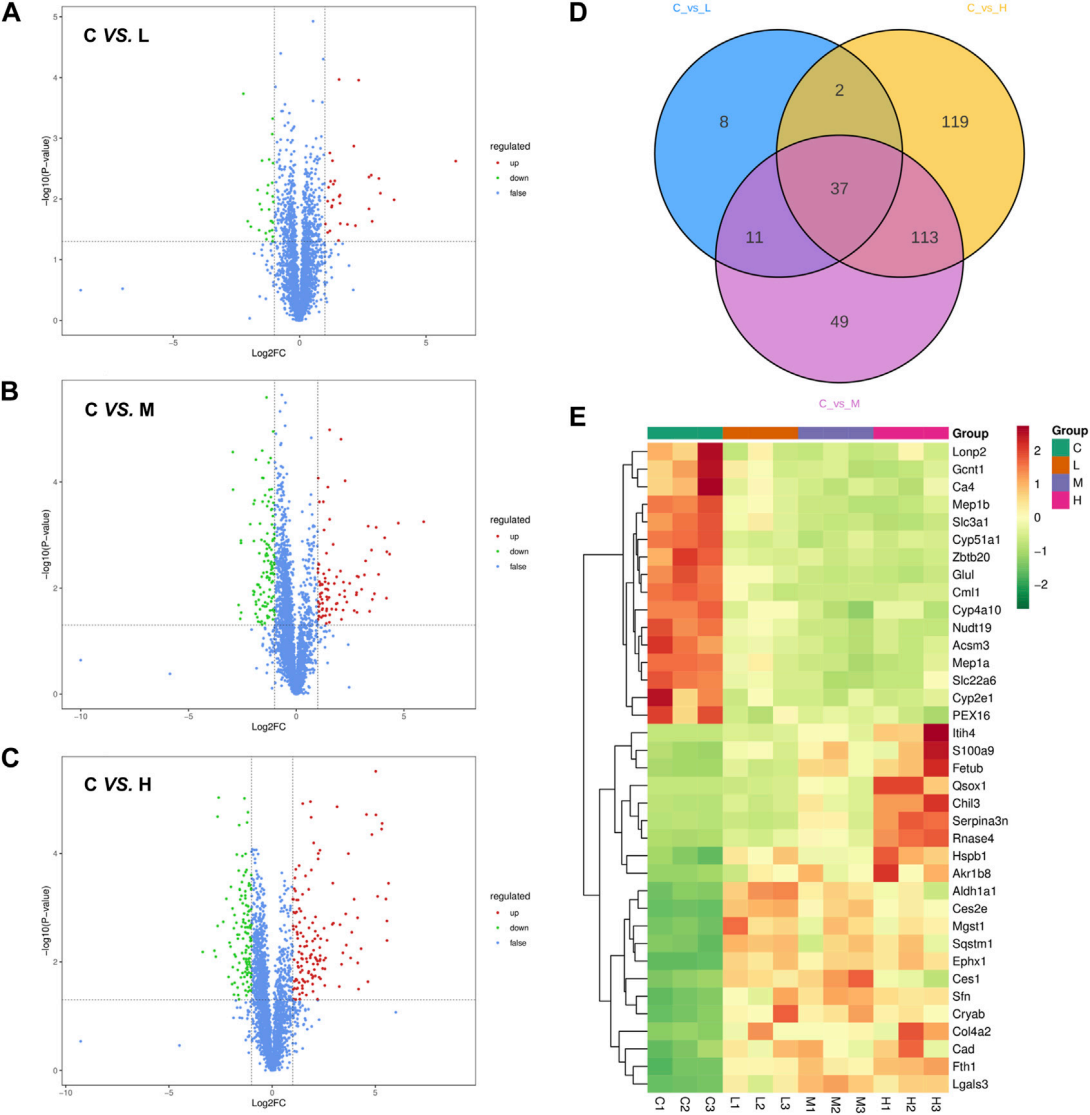
Screening for differentially expressed proteins (DEPs). **(A)** Volcano plot of protein expression changes in the control group compared with the low-dose AAI group (1.25 mg/kg/d). **(B)** Volcano plot of protein expression changes in the control group compared with the medium-dose AAI group (2.5 mg/kg/d). **(C)** Volcano plot of protein expression changes in the control group compared with the high-dose AAI group (5 mg/kg/d). Blue represents proteins with insignificant differences, red represents upregulated proteins, and green represents downregulated proteins. **(D)** Venn diagram analysis of three sets of comparisons. **(E)** Heatmap analysis of the 37 common DEPs in three sets of comparisons. Red indicates upregulation and green indicates downregulation.

The DEPs were identified according to the criteria of Fold change >2 or <0.5 and *p*-value <0.05. Compared to the control group, 31 proteins were upregulated, and 27 proteins were downregulated in the AAI low dose group (Figure 4A). Doubling of the AAI dose increased the number of upregulated proteins to 91 and the number of downregulated proteins to 119 (Figure 4B). The highest number of DEPs reached 271 (145 upregulated and 126 downregulated) in the AAI high dose group compared to the control group (Figure 4C). Intersection of these 3 sets of comparisons yielded 37 common DEPs (Figure 4D). The expression trends of these 37 proteins in each sample were presented in Figure 4E and Supplementary Table S1. These results helped to clarify the proteins that could be affected by all three doses of AAI. The expression of these proteins correlated with the occurrence and severity of AAN.

### 3.6 Bioinformatics analysis of DEPs

**FIGURE 5:**
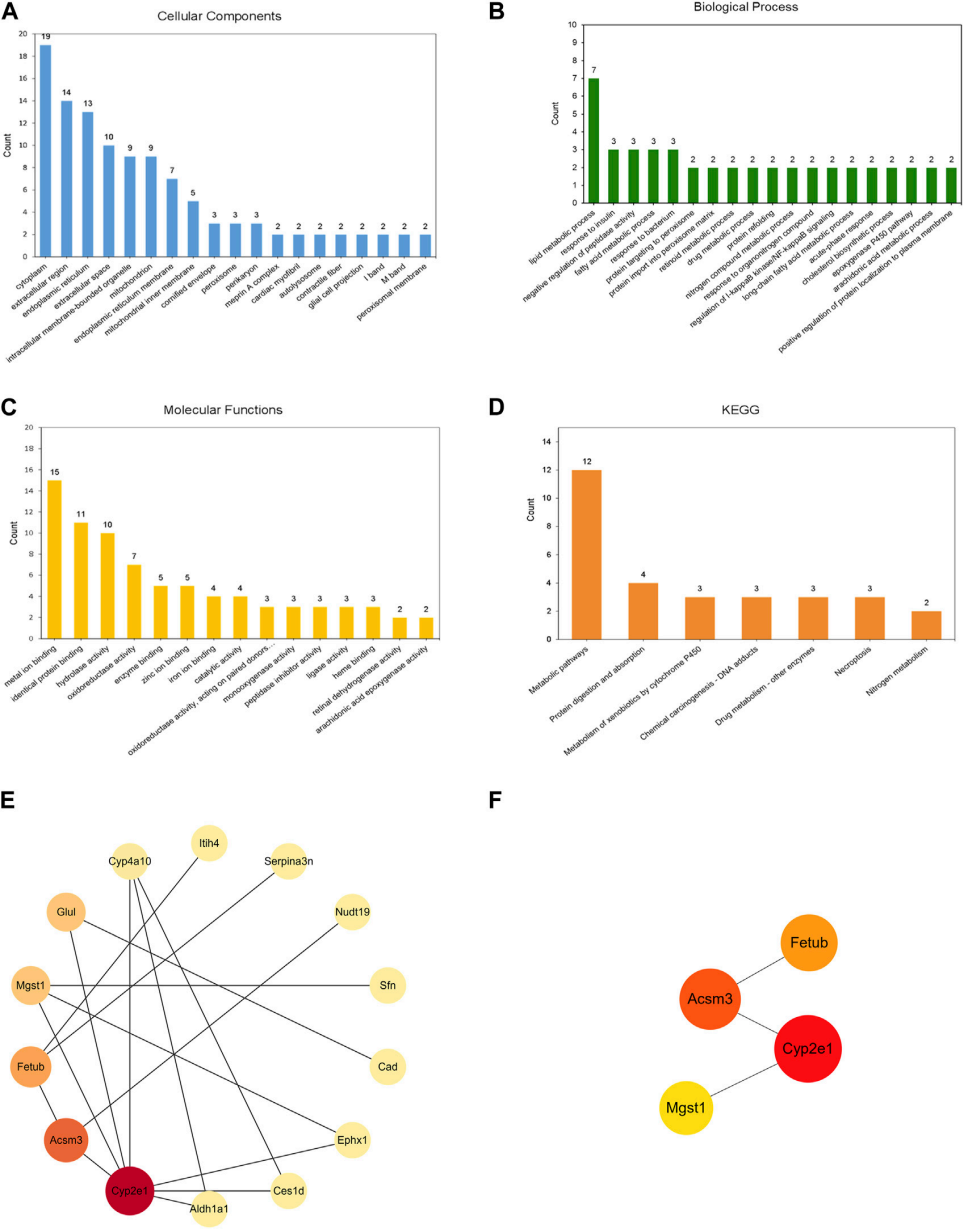
Annotations and PPI of the 37 DEPs identified in the kidneys of mice. GO analysis: **(A)** Cellular Components, **(B)** Biological Process, **(C)** Molecular Functions. **(D)** KEGG enrichment analysis **(E)** PPI network of 23 DEPs were interconnected. **(F)** PPI network of four representative proteins. PPI network was analyzed by the STRING database and the Cytoscape software. The nodes represent proteins. The size and color of the node represents the value of betweenness (a larger size and darker color indicates a higher betweenness).

GO annotation analysis of DEPs was performed to describe the role of genes and proteins in cells, to comprehensively describe the characteristics of genes and gene products in organisms. In CC, 37 DEPs were mainly concentrated in cytoplasm, extracellular region, and endoplasmic reticulum (Figure 5A). These DEPs were involved in some biological processes of BP, among which lipid metabolic process was the most important (Figure 5B). As for MF, it was mainly related to metal ion binding, identical protein binding, and hydrolase activity (Figure 5C). The diagram of KEGG pathway analysis showed seven pathways, and the most important one was metabolic pathways (Figure 5D). PPI network analysis showed that 23 DEPs were interconnected, while the other 14 DEPs showed no association. Cytoscape software was used to present the overall perspective of the relationship within 23 DEPs (Figure 5E). According to the value of betweenness, Cyp2e1, Acsm3, Fetub, Mgst1 were screened as the most important DEPs (Figure 5F).

### 3.7 Validation of key DEPs expression in mice kidneys

**FIGURE 6:**
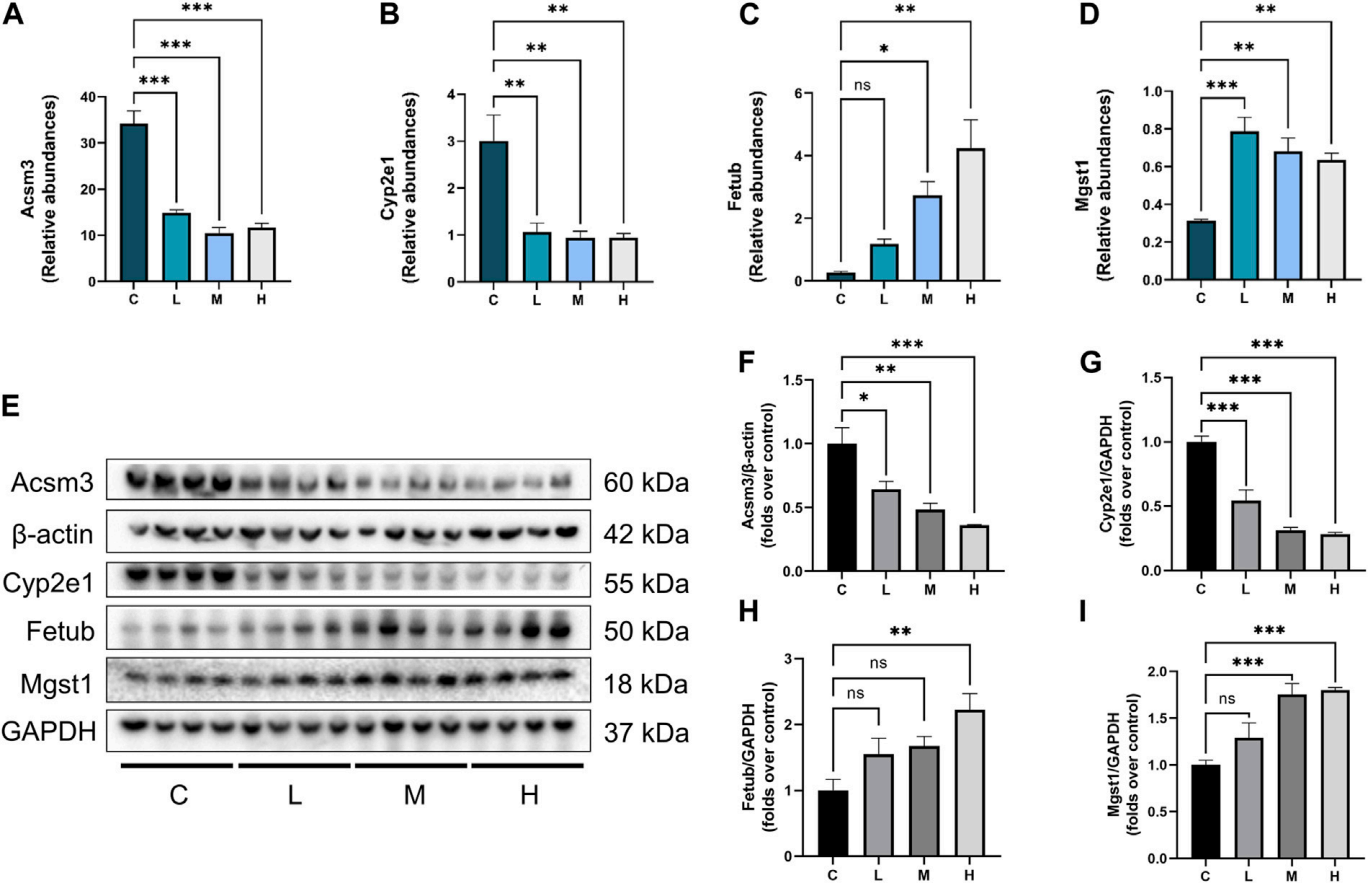
Abundances of DEPs and validation of DEPs expression. **(A–D)** Relative abundance of Acsm3, Cyp2e1, Fetub, and Mgst1 detected by proteomic analysis. **(E)** Western blot images of Acsm3, Cyp2e1, Fetub, and Mgst1. **(F–I)** Graphic presentation of kidney Acsm3 expression, normalized to β-actin; presentation of Cyp2e1, Fetub, and Mgst1 expression, normalized to GAPDH (n = 4). Data are expressed as mean ± SEM. (*p < 0.05, **p < 0.01 and ***p < 0.001 as compared to the control group).

The relative abundance of Cyp2e1, Acsm3, Fetub, and Mgst1 in the proteomics analysis was summarized in Figures 6A–D. Exposure to AAI suppressed the expression of Cyp2e1 and Acsm3 but enhanced the expression of Fetub and Mgst1. This result was further validated by Western blot analysis. As shown in Figures 6E–I, compared with the control group, the expression of Acsm3 and Cyp2e1 in the AAI groups were significantly downregulated, while the expression of Fetub and Mgst1 were upregulated in the AAI groups. It is worth noting that AAI modulated the expression levels of these proteins in a dose-dependent manner.

## 4 Discussion

In this study, we used a combination of label-free quantitative proteomics and bioinformatics annotation to investigate potential targets and pathogenesis of AAI-induced nephrotoxicity in mice. The results showed that AAI induced nephrotoxicity in a dose-dependent manner and renal protein profiles were dysregulated and exacerbated with increasing AAI dose. We screened 37 common DEPs that could be affected by all three doses of AAI. GO and KEGG analysis of the 37 DEPs showed that most of them were located in endoplasmic reticulum and mitochondria, and they were closely related to metabolism and oxidative stress. Mapping PPI networks for the 37 DEPs, we found that Acsm3 (downregulated), Cyp2e1 (downregulated), Fetub (upregulated), and Mgst1 (upregulated) were core proteins affected by AAI.

In kidney tissue, the high energy produced by mitochondria and peroxisomes through fatty acid oxidation (FAO) is an important source of energy for tubular epithelial cells. FAO inhibition could cause ATP depletion, cell death or differentiation, and lipid deposition (Meyer et al., 1997; Kang et al., 2015). Acsm3 is a member of the acyl-CoA medium chain (C4-C14) synthetase (ACSM) family (Shrestha et al., 2020), which catalyze the first step of fatty acid metabolism (Shrestha et al., 2020). The study found that exposure to AAI reduced the expression of Acsm3, which might cause defects in the fatty acid metabolic pathway, lipid accumulation and nephrotoxicity. Fetub is considered to be a novel secretory adipokine/hepatic factor that is regulated in human steatosis (significantly increased in hepatic steatosis) and mediates impaired insulin action and glucose intolerance (Meex et al., 2015; Kralisch et al., 2017). The upregulation of Fetub in AAI group suggested that AAI-induced nephrotoxicity was related to fat metabolism. Ces2e and Ces1 are carboxylesterases that play an important role in exogenous ester drug metabolism and endogenous lipid metabolism (Lian et al., 2018), and their expression levels showed an increasing trend in this study. In addition, the disruption in the expression levels of the proteins Nudt19, Lonp2, and PEX16, which are related to mitochondrial and peroxisomal productivity (Wu et al., 2018; Burkhart et al., 2019; Görigk et al., 2022), also reflected the metabolic disorders caused by AAI.

It has been demonstrated that interstitial inflammation characterized by activated mononuclear/macrophages and cytotoxic CD8^+^ and CD103^+^ T lymphocytes occurs during the progression of experimental AAN (Pozdzik et al., 2008). CD4^+^ or CD8^+^ T lymphocyte depletion has also been reported to be associated with more severe kidney injury in acute experimental AAN, suggesting a protective role for T lymphocyte in AAN (Baudoux et al., 2018). In addition to its close relationship with fatty acid metabolism, Acsm3 can also cause a significant active immune response, and its decreased expression is associated with reduced infiltration of CD8^+^ T cells, macrophages, and dendritic cells (Zhu et al., 2020). In addition, exposure to AAI increased the levels of S100a9, Itih4, Chil3, and Sqstm1 (Figure 4E). S100a9 and Itih4 have the role of regulating inflammatory response (Wang et al., 2018; Ma et al., 2021). Chil3 (Yoshimura and Oppenheim, 2011) and Sqstm1 (Tang and Kang, 2023) were reported to be activated or released in large quantities during inflammatory states. Collectively, AAI exposure placed the kidneys in an inflammatory state.

Oxidative stress occurs when the balance between oxidants and antioxidants is disrupted. AAI may interfere with free radical balance, such as the production of hydrogen peroxide (Sies, 2015). The cytochrome P450 (CYP) enzymes are membrane-bound hemoproteins that play a pivotal role in the detoxification of xenobiotics, cellular metabolism, and homeostasis (Manikandan and Nagini, 2018). It has been reported that the P450 enzyme participated in the catalytic reduction reaction leading to the biological activation of AA (Anger et al., 2020). Among the 37 DEPs we found, Cyp2e1, Cyp4a10, and Cyp51a1 all belong to this class of enzymes. Induction or inhibition of CYP enzymes is a major mechanism that underlies drug-drug interactions. In the case of Cyp2e1, it is identified as located in the proximal renal tubules and is the primary isoenzyme in the renal tubules (Liu and Baliga, 2003). Exposure of LLC-PK1 cells to AAI resulted in a decrease in Cyp2e1 content and an increase in the production of catalyzed iron and hydroxyl radicals, which triggered oxidative stress and caused damage to the kidney (Liu and Baliga, 2003). This phenomenon has also been observed in studies of myoglobin-induced acute kidney injury (Wang et al., 2014). Mgst1 is activated by oxidative stress and can strongly protect mitochondria from oxidative stress by forming many mitochondrial outer membrane proteins (Johansson et al., 2007; Johansson et al., 2010; Bräutigam et al., 2018). In this study, compared with the control group, Mgst1 in the kidneys of mice was significantly increased in the medium- and high-dose AAI groups, which might be related to the oxidative stress caused by AAI and the activation of the antioxidant defense system. Therefore, maintaining oxidation and antioxidant homeostasis may become pathogenic targets of AAI-induced nephrotoxicity.

## 5 Conclusion

In conclusion, AAI-induced nephrotoxicity was closely related to lipid metabolism disorders, inflammation, and oxidative stress. Acsm3, Cyp2e1, Fetub, and Mgst1 may be potential therapeutic targets for treating acute AAN.

## Data Availability

The datasets presented in this study can be found in online repositories. The names of the repository/repositories and accession number(s) can be found below: ProteomeXchange Consortium via the iProX. repository with the project ID: IPX0007773000.

## Abbreviations

AA, Aristolochic acid; AAN, Aristolochic acid nephropathy; ACSM, Acyl-COA medium chain (C4-C14) synthase; Acsm3, Acyl-CoA synthetase medium chain family member 3; ACSs, Acyl-COA synthetase; AKI, Acute kidney injury; ATP, Adenosine triphosphate; BP, Biological Process; BUN, Blood urea nitrogen; CC, Cellular Component; CYP450, cytochrome P450; Cyp2e1, Cytochrome P450 2E1; DEPs, Differentially expressed proteins; FAO, Fatty acid oxidation; Fetub, fetuin B; GO, Gene Ontology; KEGG, Kyoto Encyclopedia of Genes and Genomes; KOG, Clusters of orthologous groups for eukaryotic complete genomes; LC-MS/MS, Liquid chromatography-tandem mass spectrometry; MF, Molecular Function; Mgst1, Microsomal glutathione transferase 1; MS, Mass spectrometry; PAS, Periodic acid-Schiff; PPARα, Peroxisome proliferation-activated receptor α; PPI, Protein-protein interaction; SEM, Standard error of the mean; SCR, Serum creatinine.
