# Supplementary material for: Delineation of renal protein profiles in aristolochic acid I-induced nephrotoxicity in mice by label-free quantitative proteomics
Source: Front Pharmacol. 2024 May 9;15:1341854. doi: 10.3389/fphar.2024.1341854 (PMC11111959; doi:10.3389/fphar.2024.1341854)
Supplement: Supplementary file 1 [file DataSheet1.PDF]

Supplementary Figures

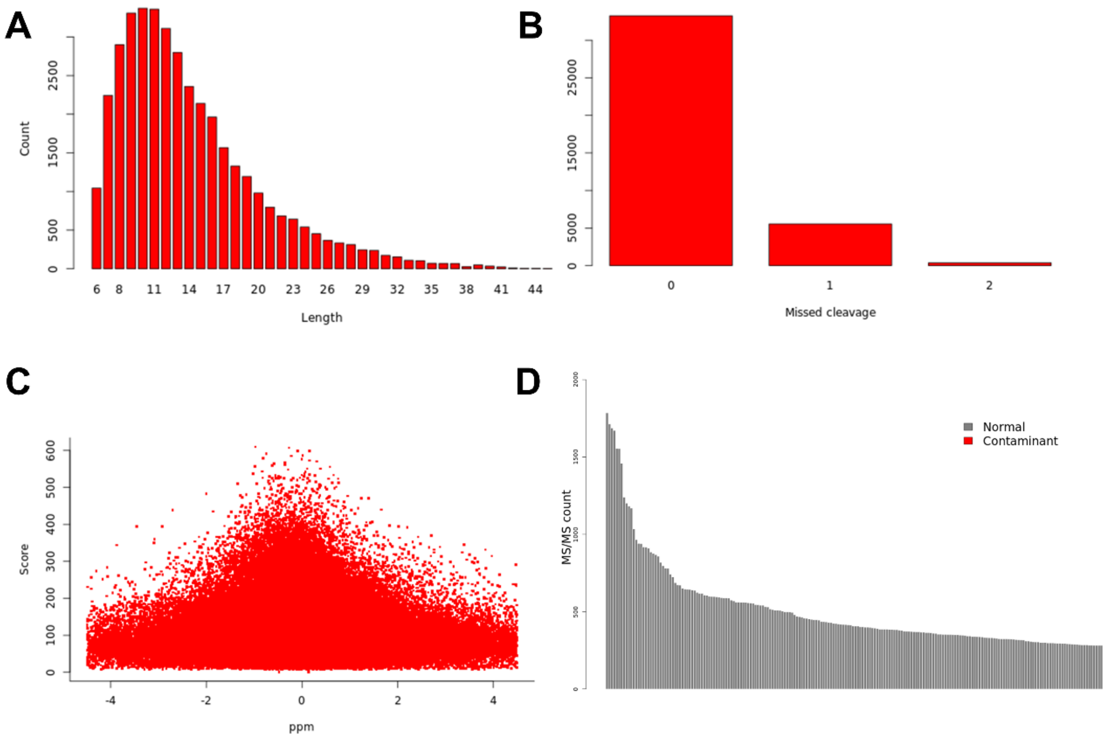

Supplementary Figure S1. Quality control data for proteomics analysis. (A) Peptide length distribution chart. (B) Distribution of missed cleavage. (C) Mass deviation distribution chart. (D) Distribution of contaminated protein profiles.

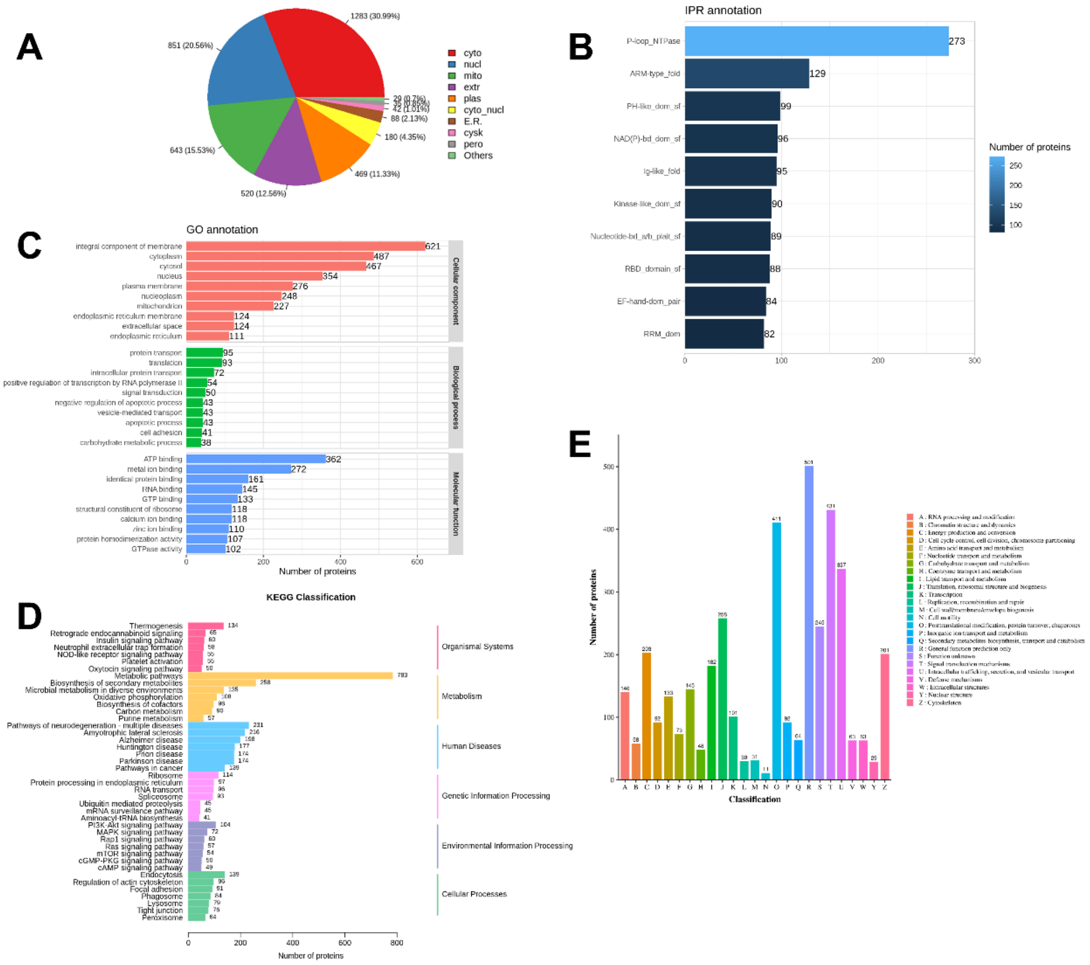

Supplementary Figure S2. Annotations of the identified proteins. (A) Sub-cellular localization. (B) Domain annotation. (C) GO annotation. (D) KEGG annotation. (E) KOG annotation.

Supplementary Table

Table S1. Expression differences between groups of the 37 common DEPs

ID	C *vs.* L		C *vs.* M		C *vs.* H	
	log2FC	*P* Value	log2FC	*P* Value	log2FC	*P* Value
Itih4	2.74	7.19E-04	3.71	5.26E-03	5.52	3.36E-02
Cad	1.89	9.95E-03	1.85	2.40E-02	2.09	2.88E-02
Chil3	1.27	1.35E-02	2.18	5.21E-02	3.70	1.31E-03
Sfn	1.28	1.94E-02	1.55	6.54E-04	1.36	5.15E-05
Cyp4a10	-1.07	4.75E-04	-1.88	4.85E-04	-1.34	5.26E-04
Col4a2	1.20	3.01E-02	1.02	9.63E-05	1.47	2.61E-02
Fth1	1.57	5.80E-05	1.72	1.03E-03	1.96	4.51E-05
Nudt19	-1.07	6.11E-04	-1.68	2.53E-04	-1.62	2.50E-04
Hspb1	1.33	3.98E-03	1.02	2.27E-03	1.68	2.26E-03
Glul	-1.58	1.18E-03	-2.94	1.63E-04	-3.36	1.65E-04
Lgals3	1.59	4.51E-03	2.08	5.69E-05	1.89	6.13E-05
Cryab	2.83	2.02E-02	2.83	2.70E-03	2.56	3.89E-04
Aldh1a1	3.19	4.98E-04	2.79	2.60E-04	2.31	1.34E-02
Mep1a	-1.09	1.58E-03	-1.80	3.80E-05	-1.59	1.10E-04
S100a9	2.21	5.78E-03	2.80	1.32E-02	3.37	6.13E-02
Akr1b8	3.14	2.13E-02	3.49	2.26E-02	4.14	2.16E-02
Cyp2e1	-1.51	2.91E-02	-1.68	2.22E-02	-1.67	2.11E-02

Gcnt1	-1.07	6.64E-02	-1.75	2.30E-02	-1.82	2.16E-02
Acsm3	-1.21	2.47E-03	-1.71	1.45E-03	-1.54	1.52E-03
Mep1b	-1.28	9.93E-04	-2.13	1.37E-04	-2.06	2.32E-04
Sqstm1	1.30	1.32E-05	1.06	1.88E-02	1.07	7.69E-03
Ca4	-1.34	7.08E-02	-1.99	3.70E-02	-1.94	4.32E-02
Ces2e	6.18	4.91E-06	5.92	7.98E-04	5.56	1.44E-04
Qsox1	1.61	3.75E-02	3.04	1.80E-02	5.11	4.55E-03
Cyp51a1	-2.23	3.65E-05	-2.56	2.03E-04	-2.37	4.27E-05
Zbtb20	-1.49	2.88E-03	-1.61	2.63E-03	-1.82	1.85E-03
Slc22a6	-1.05	3.31E-04	-1.52	1.87E-04	-1.21	1.59E-03
Ces1	2.74	1.78E-04	3.17	1.71E-03	1.90	1.40E-02
Mgst1	1.33	3.05E-03	1.12	6.56E-03	1.02	1.08E-03
Serpina3n	1.60	1.03E-02	3.53	2.61E-02	5.02	1.25E-04
Slc3a1	-1.14	4.16E-03	-1.93	2.77E-04	-2.22	2.70E-04
Pex16	-1.06	2.38E-02	-1.00	1.60E-02	-1.21	1.40E-02
Ephx1	2.34	1.20E-04	2.27	2.45E-04	2.32	2.74E-04
Lonp2	-1.19	5.17E-02	-1.44	2.71E-02	-1.13	5.10E-02
Cml1	-1.11	9.11E-04	-1.38	3.35E-06	-1.29	9.73E-06
Rnase4	1.39	2.85E-03	1.88	3.59E-03	3.15	5.31E-05
Fetub	2.14	3.92E-03	3.34	4.88E-03	3.97	1.18E-02
